# Clinical Lecture on Subacute and Chronic Rheumatism

**Published:** 1863-08

**Authors:** Henry Wm. Fuller

**Affiliations:** Physician to the Hospital


### CLINICAL LECTURE ON SUBACUTE AND CHRONIC RHEUMATISM.

*Delivered at St. George's Hospital,*

By HENRY WM. FULLER, M, D.,

*Physician to the Hospital.*

Gentlemen: In my last two lectures my observations were confined to cases of acute rheumatism, or rheumatic fever; to-day I propose to bring under your notice some varieties of disease which pass under the title of chronic rheumatism. Before doing so, however, I would say a few words respecting the cases admitted into the hospital under the title of subacute rheumatism—cases characterized by more or less swelling of the joints, with slight feverishness, acceleration of pulse, and coating of the tongue; but nevertheless not ushered in by rigors, and not marked by that degree of fever and excitement of the circulation, nor by the same amount of coating of the tongue, nor by the profuse sour-smelling perspiration and loaded urine, nor by the redness and exquisite pain and tenderness of the joints, which accompany acute rheumatism. In some hospitals, and by many practitioners, these cases are styled "acute rheumatism;" and there cannot be a doubt that the nature of the disease is the same in the one class of cases as in the other. But those of you who have watched the large number of patients whose symptoms are designated "subacute" in this hospital, must be aware how strictly they deserve the title which is given to them. They are truly "subacute," as compared with those which are styled acute; and the treatment required is in keeping with their subacute character. I will instance the case of M. H——, aged twenty-four, who was admitted into the Queen's ward on the 13th of January, 1862. Three weeks before her admission this woman was attacked with pain and swelling of the ankles, and one week before admission the right knee became swollen and painful, rendering it difficult for her to walk even across the room. When I first saw her the skin was warm and moist—not hot and perspiring; the ankles and the right knee were swollen, principally from effusion within the capsule, but were not red, and were scarcely tender to the touch; the tongue was only slightly coated; the bowels were acting regularly; the urine was somewhat cloudy; the pulse 96, soft; the catamenia were regular; the appetite was good; the heart's action was regular and its sounds were clear. She told us that the pains were much relieved by warmth; and as guaiacum is a medicine which has always appeared to me to be signally useful in such cases, I determined to administer, every six hours, the guaiacum mixture of the Pharmacopœia, with the addition of half a drachm of carbonate of potash, half a drachm of acetate of potash, and six grains of iodide of potassium. At the same time I limited her diet to fish and beef-tea. By these means I hoped to stimulate the action of the skin, to counteract acidity, to promote the action of the kidneys, and to check the further formation of the materies morbi. The iodide of potassium was given because observation has led me to believe that in subacute and chronic cases in which the articular swelling is referable principally to effusion within the capsule of the joint, this salt assists in promoting absorption of the synovial fluid, and acts beneficially as an alterative. Be this as it may, the result was satisfactory; relief was speedily obtained, and she left the hospital quite well at the end of the week (on the 19th).

In many of these cases the skin is very inactive, and when, as often happens, inactivity appears to be in great measure the cause of the continuance of the rheumatic pains, hot-baths, hot air baths, and vapor baths are essential adjuncts to the treatment. Sometimes the liver is very sluggish, as indicated by the muddiness of the complexion, the yellowness of the conjunctivæ, and the furring of the tongue; and in such instances no permanent relief will be obtained until the diet has been carefully regulated, and the liver stimulated, by repeated doses of some mercurial preparation. An excellent example of this fact has been under your notice in the case of J. B——, aged twenty-one, in Holland ward. This woman was admitted on the 10th of February, 1862, suffering rather severely from articular pain and swelling of ten days' duration. Her skin was warm and dry, not hot and perspiring, and her general symptoms were hardly such as to justify us in classing her case as one of acute rheumatism. Her symptoms were treated in accordance with their subacute character. Her skin was acted on by means of a hot-air bath, and the guaiacum mixture was ordered to be taken every six hours, with the addition of half a drachm of carbonate of potash, and half a drachm of acetate of potash. At the same time, as her complexion was muddy, the conjunctivæ yellowish, and the tongue exceedingly furred, and as the bowels were sluggish, I deemed it right to administer on alternate nights five grains of calomel and five grains of the compound extract of colocynth, followed in the morning by a senna draught, containing half an ounce of the potassio-tartrate of soda. Moreover her diet was limited to beef-tea. The bowels were freely acted on by the medicine and after each evacuation the pains were relieved, so that in a few days the swellings subsided and she was able to leave her bed. At this time (on the 16th) the complexion was still muddy, and the tongue coated, though less so than before. As she was almost free from pain, and begged for an increased allowance of food, I was induced to give her a small quantity of meat. The result was an immediate return of articular pain and swelling, which I attempted in vain to relieve by alkaline medicines and hot-air baths. No impression could be made on the symptoms until beef-tea had been again prescribed as her diet, and recourse was again had to colocynth and calomel to stimulate the secretory action of the liver and evacuate the acrid contents of the bowels. Relief was then obtained in a few days, and though her tongue had not thoroughly cleaned, and her complexion remained sallow, she was going on steadily to recovery. Some of you, however, having expressed a doubt as to whether her relapse was indeed attributable to my having given her meat and ceased to purge her, whether the recurrence of her symptoms at that particular moment was not a mere coincidence, I determined to satisfy you on this point by again ceasing to administer purgatives, and by ordering her meat for her dinner. The result was an immediate recurrence of pain and articular swelling, and a confirmation of my suspicions on the subject. Similar cases are of frequent occurrence, and are amongst the most obstinate met with in practice, if recourse is not had to repeated—I had almost said the daily—administration of purgatives.

So again, when the constitution is weak and the patient is exhausted, rheumatic medicines—falsely so called—are of little avail for the cure of the disease until the system has been invigorated by tonics. Take as an example the case of E. K., aged twenty-one, who was admitted into the Roseberry ward, on the 18th of last November. She was pale, weakly and out of health when she was attacked, three weeks before her admission to the hospital, with wandering pains in the limbs and occasional swelling of the joints. On admission there was swelling of both knees and the wrist, but no redness and very little tenderness; the skin was warm and moist, the tongue clean, the bowels were regular, the urine was clear, the pulse 84, soft and weak. The guaiacum mixture was ordered to be taken every six hours, with the addition of a drachm of the ammoniated tincture of guaiacum, a scruple of carbonate of potash, and six grains of iodide of potassium. This treatment with some trifling alteration, was continued until the 3d of December, when, as little or no improvement had taken place, I substituted the cinchona draught for the guaiacum mixture, and continued the other medicines as before. A marked change occurred immediately. Within a few days her aspect became more healthy; by the 8th the swellings had disappeared; and by the 12th there were no longer any pains in the joints. On the 15th she left the hospital. Let me beg of you, then, to the indications which point to the necessity for tonics. Knowing, as I do, from long experience, how frequently their exhibition is necessary, I am nevertheless too apt, as in this case, to overlook the symptoms which call for their administration. Much more will this be so with you, if you do not bear the fact constantly in mind. A clean tongue and clear urine, when met with coincidently with a cool, moist skin, or a clammy perspiration, are symptoms which denote a languid state of the system, and demand the aid of tonics; and although the form of tonic required may vary in different cases, you will rarely succeed in relieving your patient if you fail to impart tone and vigor to his system. You will see this fact constantly illustrated in practice; and now that your attention has been specially directed to it, you alone must bear the blame if you do not recognize the necessity when it occurs, and act on the suggestions I have thrown out.

One word before quitting this subject on the frequency of inflammation of the heart in connection with "acute" and "subacute" rheumatism. The statistics which I collected in the wards of this hospital during the period of my registrarship* show that whereas the heart is damaged to a greater or less extent in 1 out of every 2.06 of the cases designated "acute" in our wards, it suffers only in 1 out of every 6.65 of the cases which are styled "subacute;" and if this latter class were still

* See my work on Rheumatism, Rheumatic Gout, and Sciatica, 3d edition, pp. 260—284.

further extended, and were made to include examples of rheumatic gout, and cases of rheumatism characterized by severe pain, but unaccompanied by articular swelling or febrile disturbance, the proportion of cases in which the heart would become damaged would fall to 1 in 36.25 cases. In a therapeutical point of view, this difference in the liability to heart affection, according as the rheumatism assumes one type or another, is a matter of grave importance, as proving how much more energetic should be our treatment in one case than in another, and how much more watchful we should be for the accession of cardiac mischief. But its practical bearing on the statistics of heart disease is even more important, for you will readily understand how much more favorable any particular mode of treatment may be made to appear, in reference to cardiac complications, if cases of subacute and chronic rheumatism, and cases of rheumatic gout, are included under the head of acute rheumatism, than they would be if the term acute rheumatism was restricted as it is in the wards of St. George's Hospital.

We will now pass on to the consideration of cases of so-called chronic rheumatism. If the term chronic rheumatism were to be limited to cases in which pains in the limbs resulted from the presence of the materies morbi which gives rise to true rheumatism as typefied by rheumatic fever, my conviction is that you would meet with comparatively few examples of this form of disease. Experience shows that the sufferers from rheumatic fever are usually exempt from chronic rheumatism, and that if, as sometimes happens, they do have pains in the limbs, the attack soon subsides under appropriate treatment, or else runs into rheumatic fever. Further, the disease bears the impress of true rheumatism from its very commencement. The patients are pale or sallow, the tongue is coated, the urine shows a tendency to deposit lithates, the pains manifest a strangely migratory character—shifting repeatedly from limb to limb, and alkaliss, combined it may be with tonics, afford almost immediate relief. But, as I told you at the beginning of my lecture, many varieties of disease are comprised under the title of chronic rheumatism; and you will readily understand that if this be so—if cases differing not merely in their general features, but in their mode of origin and essential cause, are classed together under the same title—the treatment which may check the progress of one case may fail to exercise the slightest influence over the course of another. And so in practice it is found to be. It is not that a remedy is efficacious in some particular form of rheumatism in one person and fails to relieve the same form of rheumatism in another; on the contrary, the medicine which proves remedial in one person will usually eradicate the same form of the complaint in another. So generally does this hold good, that it forms an almost unanswerable argument in favor of a plurality of poisons; and I do not hesitate to state my conviction that many cases of so-called rheumatism have nothing in common with true rheumatism as typefied by rheumatic fever, but arise from the presence of totally different materies morbi, and, consequently, require a totally different treatment.

Let me take a few examples of some of the more common and more obstinate forms of so-called chronic rheumatism. P. D——, aged fourteen, a cachectic boy, who had suffered for some months from cough and general debility, resulting, as it appeared, from insufficient nourishment and general neglect at home, was admitted into the Fuller ward on the 16th of November. His principal complaint was of pains in his limbs and finger-joints, and occasional swelling of the knees. These had troubled him about twelve months, and had latterly become so severe as to cause him to disregard his cough. He had been under medical treatment above two months, but he had not obtained relief. His aspect was cachectic; his skin cold and clammy; the circulation languid, so that his hands and fingers were of a purplish color; the pulse was 90 and weak; the tongue clean; the urine clear; the bowels were reported regular; the appetite was indifferent. Here, then, was a case—an obstinate case of so-called rheumatism—of rheumatism so obstinate that it had resisted all means employed to get rid of it above twelve months! Yet what single symptom of true rheumatism did it present, except pain and occasional swelling of the joints? The tongue was clean, the bowels were regular, the urine was clear, the skin cool; there was no feverishness, no evidence of the presence of that acidity by which true rheumatism is characterized. But as there were pains in the limbs and joints, the malady was called rheumatism, and as rheumatism I doubt not it had been treated, for the boy had not only taken medicines, but had had repeated warm baths. The result I have just told you: he had failed to obtain the slightest relief, and at length was brought to the hospital. No wonder that treatment directed against rheumatism should have failed so signally: for the boy was not suffering from that disease, but from irritation and pain in his joints resulting from a cachectic condition of his blood altogether distinct from that which exists in rheumatism. Those of you who are familiar with the features of gout, or who have noted the severe aching pains in the limbs by which an attack of typhus fever is ushered in; the intense pain in the loins and oftentimes in the limbs also which accompanies an attack of smallpox, and pains in the limbs, with occasional effusion into the capsules of the joints so commonly met with in cases of glanders, and in the cases of starvation which sometimes seek a refuge in our wards, need not be reminded that other agencies besides the rheumatic poison may excite pains in the limbs and swelling of the joints, and will admit that the only so-called rheumatic symptom from which this boy was suffering, may and often does arise quite independently of any true rheumatic influence. The only indications for treatment in this case—the indications which any one must have seen who could divest himself of preconceived ideas as to rheumatism—were the cachectic condition of the system, the cold clamminess of the skin, the feebleness of the circulation, and the want of appetite. With the view of meeting these difficulties I gave him a five-grain compound rhubarb pill to make sure of a proper emptying of the bowels, and prescribed a draught to be taken three times a day, containing a drachm of the syrup of iodide of iron, an ounce of the infusion of quassia, and half an ounce of cod-liver oil. At the same time I urged him to try to eat meat, and ordered him the ordinary diet of the hospital and a pint of porter. Strange treatment for his rheumatism! Yet it proved eminently successful, whereas the orthodox hot baths and rheumatic remedies had failed. Within a fortnight his aspect had improved, he had gained strength, and the pains were greatly relieved. At the expiration of three weeks, his pains having wholly ceased, and his strength and health becoming daily more satisfactory, he was made an out patient of the hospital. Before he left I directed him to continue his medicine for at least another month, on the ground that in all these cases it is necessary, not only to relieve the pains, but to guard against their recurrence by a lengthened course of tonics.

The case of E. C———, a married woman, aged thirty, whom you will remember in the Queen's ward, is an example of another form of malady which is styled rheumatism; but has nothing, except pain, in common with true rheumatism. She had usually enjoyed good health; but had suckled her youngest child above fourteen months, and had felt weak and languid ever since. Whilst in this state she was attacked with severe pain, extending from the right knee down to the toes. This pain commenced three months before she applied to the hospital, and although she had been under medical treatment above two months, and had not only taken a large quantity of medicine, but had employed a variety of embrocations, she had failed in obtaining the slightest relief. She described the pain as of a burning character, and as being aggravated in paroxysms at irregular intervals. It was not accompanied by redness or swelling, but during a paroxysm there was decided increase of heat in the part. There had not been pain in other part of the body. When I first saw her she was pale and sallow; the skin was natural; tongue coated; bowels reported regular, and urine clear; the pulse was 84, regular, but weak; the catamenia, were regular but too profuse. Now, without stopping to inquire into the pathology of this form of disease, which, if not recognized as distinct from true rheumatism is usually little amenable to remedies, I would direct your attention to the clinical history of the case under consideration as bearing upon the treatment to be adopted. In the first place, the symptoms, if carefully examined, do not bear the impress of true rheumatism. There was no previous history of rheumatism, and no turbidity of the urine; there was no redness, no swelling, no shifting of the pain from limb to limb, as is almost invariably observed in rheumatism. But there was coating of the tongue, feebleness of the pulse, and distinct paroxysmal increase of pain, and the severity of the pain was far in excess of what the local symptoms appeared to indicate. In short, the pain was evidently of a neuralgic character, and the disposition to it was probably engendered by debility and exhaustion, resulting from her having suckled her child above fourteen months. Such, at least, was my view of the case, and I ordered her accordingly the ordinary diet of the hospital, with a pint of porter, and prescribed the following medicines: Colocynth and calomel pill, ten grains, at bedtime. Tincture of aconite, six minims; bisulphate of quinine, five grains; dilute sulphuric acid, twelve minims; water to an ounce and a half; every six hours. By this means I hoped to get rid of any offending matter which might have accumulated in the bowels, and given rise to the coating of the tongue, and to subdue the exalted sensibility of the nerves with which her suffering was manifestly connected. The result proved that my view was correct; for within four days the pain in the leg was much relieved, and at the expiration of a week she left the hospital, having thoroughly got rid of the pain, which for three months had not ceased to trouble her.

